# ATR Inhibitor Synergizes PARP Inhibitor Cytotoxicity in Homologous Recombination Repair Deficiency TK6 Cell Lines

**DOI:** 10.1155/2023/7891753

**Published:** 2023-02-06

**Authors:** Rakkreat Wikiniyadhanee, Tassanee Lerksuthirat, Wasana Stitchantrakul, Sermsiri Chitphuk, Shunichi Takeda, Donniphat Dejsuphong

**Affiliations:** ^1^Program in Translational Medicine, Chakri Naruebodindra Medical Institute, Faculty of Medicine Ramathibodi Hospital, Mahidol University, Bang Phli, Samutprakarn 10540, Thailand; ^2^Research Center, Faculty of Medicine Ramathibodi Hospital, Mahidol University, Bangkok 10400, Thailand; ^3^Shenzhen University School of Medicine, Shenzhen, Guangdong 518054, China; ^4^Department of Radiation Genetics, Medical School, Kyoto University, Kyoto 606-8501, Japan

## Abstract

The inhibition of poly(ADP-ribose) polymerases (PARPs) and ataxia telangiectasia and Rad3-related (ATR) would be an alternative approach for cancer treatments. The aim of this study is to investigate the synergy of the different combinations of PARP inhibitors (olaparib, talazoparib, or veliparib) and ATR inhibitor AZD6738. A drug combinational synergy screen that combines olaparib, talazoparib, or veliparib with AZD6738 was performed to identify the synergistic interaction, and the combination index was calculated to verify synergy. TK6 isogenic cell lines with defects in different DNA repair genes were used as a model. Cell cycle analysis, micronucleus induction, and focus formation assays of serine-139 phosphorylation of the histone variant H2AX demonstrated that AZD6738 diminished G2/M checkpoint activation induced by PARP inhibitors and allowed DNA damage-containing cells to continue dividing, leading to greater increases in micronuclei as well as double-strand DNA breaks in mitotic cells. We also found that AZD6738 was likely to potentiate cytotoxicity of PARP inhibitors in homologous recombination repair deficiency cell lines. AZD6738 sensitized more genotypes of DNA repair-deficient cell lines to talazoparib than to olaparib and veliparib, respectively. The combinational approach of PARP and ATR inhibition to enhance response to PARP inhibitors could expand the utility of PARP inhibitors to cancer patients without BRCA1/2 mutations.

## 1. Introduction

Genomic instability is one of cancer hallmarks [1]. Cancer cells encounter high levels of replication and oxidative stresses; however, most cancer cells are able to circumvent these stresses to survive [2, 3]. Over decades, poly(ADP-ribose) polymerases (PARPs) have been a promising target in cancer therapy [4]. PARP inhibitors take an advantage of high levels of replication and oxidative stresses, as well as an impairment of DNA repair pathways in cancer cells to further increase genomic instability [5]. The previous study has shown that the capacity of PARP inhibitors to trap PARPs onto DNA is related with the cytotoxicity of PARP inhibitors [6]. The inhibition of PARPs leads to single-strand DNA (ssDNA) breaks that require ataxia telangiectasia and Rad3-related (ATR) to initiate the repair machinery [7]. Unrepaired ssDNA breaks due to PARP inhibition can be converted into double-strand DNA (dsDNA) breaks during DNA replication [8]. Generally, the accumulation of dsDNA breaks causes an activation of G2/M checkpoint to prevent damaged cells from entering mitosis [9]. Replication-induced dsDNA breaks are faithfully repaired by homologous recombination repair (HRR) mechanism that requires BRCA1/2; therefore, cancer cells deficient in functional BRCA1 or BRCA2 are extremely sensitive to PARP inhibitors, such as olaparib [10, 11]. Currently, four PARP inhibitors: olaparib, niraparib, rucaparib, and talazoparib, have been approved by the European Medicines Agency and by U.S. Food and Drug Administration. Those PARP inhibitors are the drugs for specific groups of breast, ovarian, and pancreatic cancer patients with BRCA1/2 germline mutations [12, 13]. Many PARP inhibitors are still in the development for treatments of cancers, such as veliparib [14], rucaparib [15], niraparib [16, 17], and talazoparib [16, 18]; moreover, some of them are undergoing clinical trials with focus on combinational regimens of PARP inhibitors with chemotherapy or radiation [19].

It has been hypothesized that DNA damage response (DDR) pathways mediated by ataxia-telangiectasia mutated (ATM) and ATR are responsible for cell survival in response to PARP inhibitors [20]. There is evidence suggesting that DDR inhibitors combined with PARP inhibitors can improve cancer killing and conquer acquired resistance. For instance, PARP and ATR inhibition sensitizes BRCA1-deficient cells that are resistant to olaparib [21]. Moreover, in clinical trials, olaparib has shown the promising results in metastatic castration-resistant prostate cancer patients with mutations in other genes associated with the HRR pathway [22, 23]. Thus, targeting the DDR pathway is one of potential targets to treat many types of cancer that have defects in DNA repair system [24]. It has been suggested that PARP inhibitors could be potentially used for other indications, such as colon and lung cancer with mutations in ATM [25, 26]. The inhibitors of DDR have been extensively developed, including ATM, ATR, and DNA-dependent protein kinase (DNA-PK) to deal with cancer cells. These inhibitors potentially target cancer cells with high mutational burden. This concept is also known as synthetic lethality [27–29]. Therefore, targeting DDR and DNA repair pathways using combination strategies could be rationally applied to treat cancers. Theoretically, cytotoxicity of PARP inhibitors would be potentiated by targeting the G2/M checkpoint in order to allow cells to undergo mitosis despite the presence of DNA lesions [30]. The inhibition of cell cycle kinases, such as ATR, could amplify the cytotoxicity of PARP inhibitors [31]. For example, PARP and ATR inhibition increases genomic instability and cell death in ATM-deficient cancer cells [20]. Furthermore, dose scheduling optimization of PARP inhibitors combined with ATR inhibitors can improve anticancer activity with minimal systemic toxicity [32]. The combination of PARP inhibitors with chemotherapy or other agents brings about significantly increased toxicity, so the clinical trials are required [33]. In addition, a complete spectrum of potentially effective combinations and biomarkers that predict the response to the combination of PARP and ATR inhibitors has not been fully elucidated.

In this study, we investigated effects of ATR inhibitor AZD6738 in potentiating the cytotoxicity of three PARP inhibitors using different DNA repair-deficient TK6 isogenic cell lines as a model. We show that combining PARP inhibitors with AZD6738 contributes to an increase in replication-associated DNA damage and micronucleus induction. We also reveal that a synergistic effect is likely to be observed in homologous recombination repair deficiency cell lines. AZD6738 specifically synergizes some PARP inhibitors in particular DNA repair-deficient cell lines, providing rationale for scrutinizing synergistic interactions between PARP and ATR inhibitors in immortalized or cancer cell lines that have defects in another HRR-associated gene.

## 2. Materials and Methods

### 2.1. Drugs

Three PARP inhibitors and one ATR inhibitor were purchased from the MedChemExpress, LLC: olaparib (cat. no. 763113-22-0), talazoparib (cat. no. 1207456-00-5), veliparib (cat. no. 912444-00-9), and AZD6738 (cat. no. 1352226-88-0). All drugs were reconstituted and stored in dimethyl sulfoxide (DMSO, cat. no. 67-68-5; PanReac AppliChem). Some *in vitro* experiments required mock treatment at specific concentrations of DMSO if the final concentration of DMSO was higher than 0.1%.

### 2.2. Cells and Cell Culture

Human lymphoblastoid TK6-derived wild-type (*WT*), tumor suppressor p53-binding protein 1 knockout (*53BP1^−/−^*), RAD54 knockout (*RAD54^−/−^*), ATM knockout (*ATM^−/−^*), and breast cancer type 1 susceptibility protein conditional knockout (*BRCA1^AID/AID^*) human cell lines [34–36] were provided from Professor Shunichi Takeda (Department of Radiation Genetics, Graduate School of Medicine, Kyoto University). All cell lines were cultured in RPMI-1640 medium (cat. no. 1IVG2-11875-093; Invitrogen) supplemented with 5% horse serum (cat. no. 1IVG3-16050-122; Invitrogen), 200 mg/ml sodium pyruvate (cat. no. 113-24-6; Sigma-Aldrich), and 100 U/ml penicillin/streptomycin (cat no: 1IVG7-15140-122) at 37°C with 5% CO_2_. The *BRCA1^AID/AID^* cell line was engineered by tagging the miniauxin-induced degron (mAID) into the C-terminal end of the *BRCA1* gene [34]. To conditionally disrupt *BRCA1* gene expression for *in vitro* experiments, *BRCA1^AID/AID^* cells were cultured in the medium containing 250 *μ*M 3-indoleacetic acid (auxin, cat. no. 87-51-4; Sigma-Aldrich) for 2 h before exposing to drugs and for entire experiments.

### 2.3. Cell Cycle Analysis

Asynchronized cells were treated with indicated PARP inhibitor (0.2 *μ*M olaparib, 2 nM talazoparib, or 6 *μ*M veliparib) alone, 0.45 *μ*M AZD6738 alone, or combinations of each PARP inhibitor and AZD6738 for 20 h. Cells were fixed with 4% paraformaldehyde for 10 min at room temperature and permeabilized with 0.3% Triton-X (cat. no. 9002-93-1; Sigma-Aldrich) for 10 min at room temperature. DNA content was stained by propidium iodide (cat. no. 25535-16-4; Sigma-Aldrich) and assessed by flow cytometry on a Navios flow cytometer and Kaluza analysis 2.1 software (Beckman Coulter, Inc.). At least 10,000 events were analyzed per sample.

### 2.4. Micronuclei and gH2AX Focus Formation Assays

PARP inhibitor (0.2 *μ*M olaparib, 2 nM talazoparib, or 6 *μ*M veliparib) alone, 0.45 *μ*M AZD6738 alone, or combinations of each PARP inhibitor and AZD6738 were used to treat asynchronized cells for 20 h before harvesting. Cells were harvested onto glass slides using a Shandon Cytospin® 4 cytocentrifuge (Thermo Fisher Scientific, Inc.). A cell staining protocol for micronuclei and serine-139 phosphorylation of the histone variant H2AX (gH2AX) focus formation assays has been described previously [37]. Briefly, cells were subsequently fixed with 4% paraformaldehyde for 10 min, permeabilized with 0.5% Triton-X for 10 min, blocked with an Intercept® blocking buffer (cat. no. 190425; Li-Cor, Inc.) for 1 h, and incubated with rabbit anti-phospho-histone H2AX (Ser139) antibody (cat. no. 9718S, lot. no.17; 1 : 1000; Cell Signaling Technology, Inc.) overnight at 4°C. Then, cells were incubated with donkey anti-rabbit IgG (H+L) highly cross-adsorbed secondary antibody, Alexa Fluor 594 (cat. no. 1IVM-A21207, lot no. 1987293; 1 : 500; Invitrogen) for 1 h and counterstained with Hoechst 33342 (cat. no. 1IVM-H3570, lot no.1779684; Invitrogen). Coverslips were mounted onto glass slides using Mowiol® 4-88 (cat. no. 9002-89-5; Sigma-Aldrich). All samples stained by fluorescent dyes were observed using a fluorescence microscope (Nikon Corporation) with the same exposure time and intensity. NIH ImageJ v1.51k [38] was used to process and visualize data acquired from the microscope. gH2AX foci were observed in mitotic cells with condensed chromosomes before a metaphase plate was formed. Both micronuclei and gH2AX foci were manually counted. At least 500 nuclei and 70 mitotic nuclei were observed for micronuclei and gH2AX focus formation assays, respectively.

### 2.5. Sensitivity Assay and Combination Index Calculation

Asynchronized cells were seeded into 96-well plates at a final concentration of 30,000 cell/ml in 200 *μ*l of medium per well. Cells were treated with serial dilutions of indicated PARP inhibitor (olaparib, talazoparib, or veliparib) alone, AZD6738 alone, or combinations of each PARP inhibitor and AZD6738 for 72 h. For the combination of PARP inhibitor and AZD6738, the drug combinations were serially plated with constant ratio concentration in 96-well plates. Cell viability was determined by CellTiter-Glo® Luminescent Cell Viability Assay (cat. no. G7572; Promega Corporation). The combination index (CI) values at IC_50_ were calculated using CompuSyn version 1.0 (ComboSyn, Inc.).

### 2.6. Statistical Analysis

Statistical analysis was performed using GraphPad Prism software version 8 (GraphPad Software, Inc.). All results except gH2AX focus formation assay and CI calculation were expressed as averages and standard deviations, and *p* values were calculated using Student's *t*-test. For gH2AX focus formation assay, medians were shown, and *p* values were calculated using the Mann-Whitney test. For CI calculation, averages and standard errors were displayed.

## 3. Results

### 3.1. ATR Inhibitor AZD6738 Attenuates G2/M Arrest Induced by PARP Inhibitors

**Figure 1:**
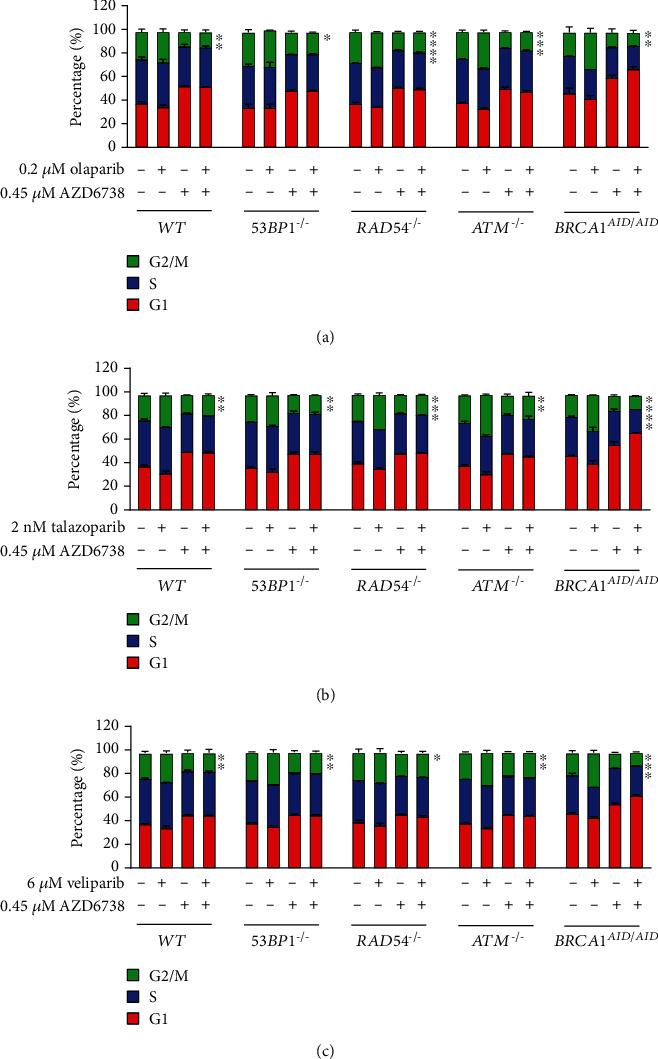
ATR inhibition facilitates the cell cycle progression of cells containing PARP inhibitor-induced DNA damage. The proportion of cells in G1, S, and G2/M of the cell cycle of indicated genotypes exposed to PARP inhibitor ((a) olaparib, (b) talazoparib, and (c) veliparib) and/or AZD6738. Averages and standard deviations of three independent experiments are displayed. The statistical difference of proportions of cells in G2/M phases between PARP inhibitor with and without AZD6738 treatments within the same genotype was calculated using Student's *t*-test. ^∗^*p* < 0.05; ^∗∗^*p* < 0.005; ^∗∗∗^*p* < 0.001; ^∗∗∗∗^*p* < 0.0001.

To investigate the effects of different PARP inhibitors with and without AZD6738 on G2/M cell cycle arrest, different DNA repair-deficient isogenic cell lines were treated with PARP inhibitor alone, AZD6738 alone, and the combination of PARP inhibitor and AZD6738. The percentages of cells in G2/M phases were quantified (Figure 1). The treatment of olaparib, talazoparib, or veliparib alone was likely to increase the G2/M population in *WT* and the other DNA repair-deficient cell lines. The increased G2/M population was obviously observed in *ATM^−/−^* and *BRCA1^AID/AID^* cell lines treated with PARP inhibitor alone. These data suggest that olaparib, talazoparib, and veliparib induce G2/M arrest. In contrast to PARP inhibition, the treatment of AZD6738 alone decreased the G2/M population in every indicated genotype compared to control treatments. Moreover, combining olaparib, talazoparib, or veliparib with AZD6738 significantly decreased the G2/M population in every indicated genotype compared to PARP inhibitor treatment alone (Figure 1). These data indicate that ATR inhibition with AZD6738 suppresses the G2/M checkpoint activation induced by PARP inhibitors.

### 3.2. ATR Inhibitor AZD6738 Amplifies the Effects of PARP Inhibitors on Micronucleus Induction

**Figure 2:**
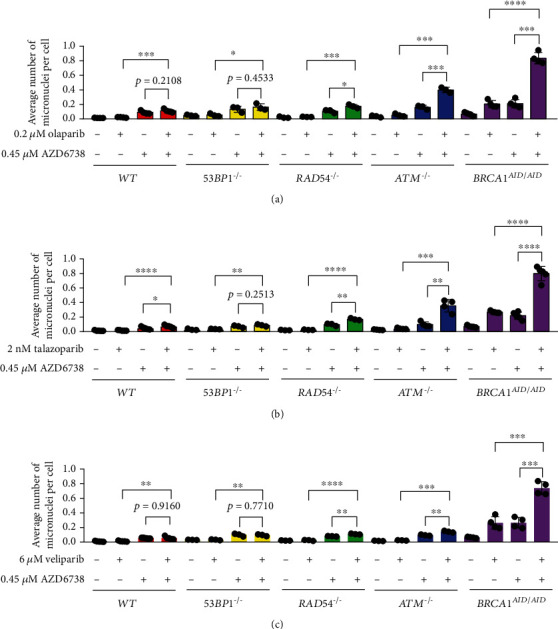
The combination of PARP and ATR inhibitors increases a number of micronuclei. Average numbers and standard deviations of micronuclei of indicated genotypes exposed to PARP inhibitor ((a) olaparib, (b) talazoparib, and (c) veliparib) and/or AZD6738 are displayed. At least three independent experiments were performed. The statistical difference of numbers of micronuclei between treatments within the same genotype was calculated using Student's *t*-test. ^∗^*p* < 0.05; ^∗∗^*p* < 0.005; ^∗∗∗^*p* < 0.001; ^∗∗∗∗^*p* < 0.0001.

The mechanism underlying the effects of ATR inhibitor AZD6738 when combining with PARP inhibitor was investigated by observing a number of micronuclei and a number of gH2AX foci in mitotic nuclei. To investigate whether AZD6738 combined with PARP inhibitor leads to an increased number of micronuclei, DNA repair-deficient cell lines were treated with PARP inhibitor alone, AZD6738 alone, and the combination of PARP inhibitor and AZD6738. Either PARP inhibitors or AZD6738 induced the formation of micronuclei. Furthermore, combining olaparib, talazoparib, or veliparib with AZD6738 notably enhanced the formation of micronuclei in *RAD54^−/−^*, *ATM^−/−^*, and *BRCA1^AID/AID^* cell lines, but not in *WT* and *53BP1^−/−^* cell lines (Figure 2). These results emphasize that the inhibition of ATR with AZD6738 promotes G2/M checkpoint override, resulting in an increased number of micronuclei.

### 3.3. ATR Inhibitor AZD6738 Aggravates PARP Inhibitor-Induced DNA Damage in Mitotic Cells

**Figure 3:**
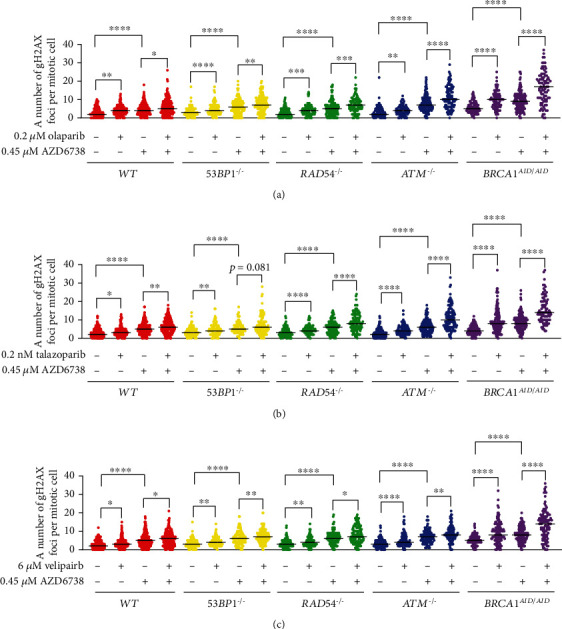
The combination of PARP and ATR inhibitors increases an amount of DNA damage in mitotic cells. A number of gH2AX foci per mitotic nucleus of indicated genotypes treated with PARP inhibitor ((a) olaparib, (b) talazoparib, and (c) veliparib) and/or AZD6738 are shown. Black lines indicate medians. The statistical difference of a number of gH2AX foci per mitotic nucleus between treatments within the same genotype was calculated using the Mann-Whitney test. ^∗^*p* < 0.05; ^∗∗^*p* < 0.005; ^∗∗∗^*p* < 0.001; ^∗∗∗∗^*p* < 0.0001.

It was hypothesized that the increased number of micronuclei in combination treatments of PARP inhibitor and AZD6738 is a result from ATR inhibition that allows cells to undergo mitosis with PARP inhibitor-induced DNA lesions. To investigate whether the combination of PARP inhibitor and AZD6738 increases an amount of DNA damage in cells entering mitosis, DNA repair-deficient cell lines were treated with PARP inhibitor alone, AZD6738 alone, and the combination of PARP inhibitor and AZD6738. The inhibition of either PARP or ATR alone contributed to an increased number of gH2AX foci in mitotic nuclei. In addition, combining olaparib, talazoparib, or veliparib with AZD6738 resulted in a significantly further increase in gH2AX foci in mitotic nuclei of every indicated genotype. However, the enhanced effect of each individual PARP inhibitor amplified by AZD6738 on a number of gH2AX foci in mitotic nuclei was clearly noticed in the *BRCA1^AID/AID^* cell line (Figure 3). Collectively, combining PARP inhibitor with AZD6738 leads to the presence of increased DNA damage at mitotic entry.

### 3.4. ATR Inhibitor in Combination with PARP Inhibitor Is Synergistic

**Figure 4:**
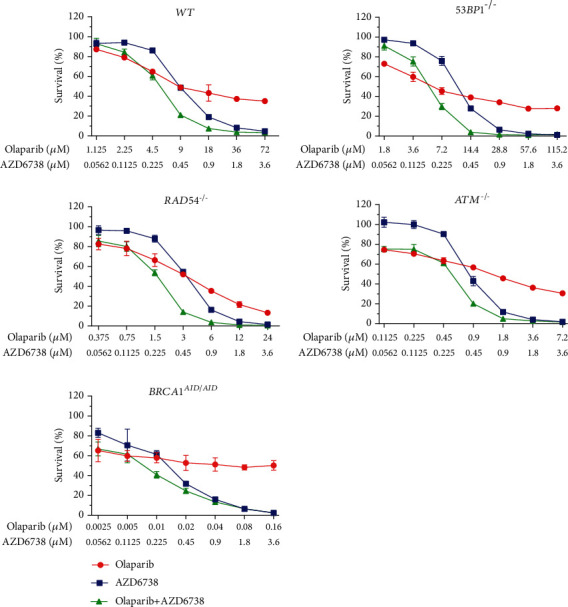
The combination of olaparib and AZD6738 decreases the cell survival. Percent survivals are displayed for each indicated genotype treated with olaparib and/or AZD6738. The experiments were performed in triplicate with two independent experiments. Averages and standard deviations are shown.

**Figure 5:**
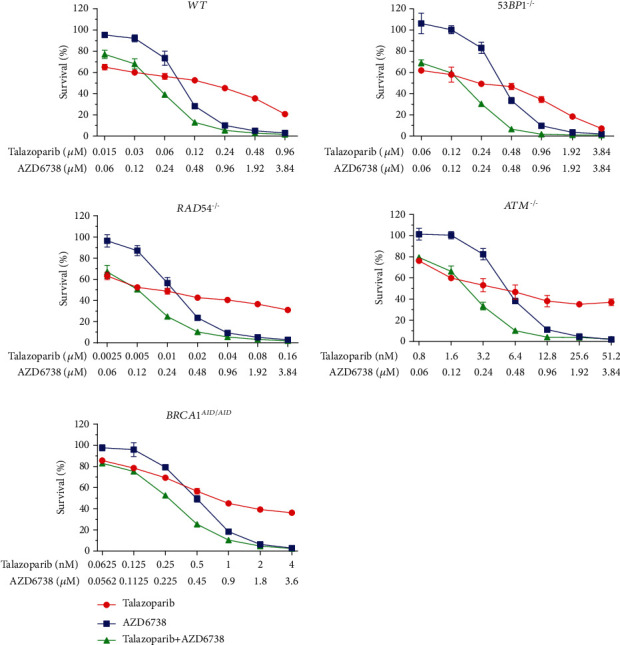
The combination of talazoparib and AZD6738 decreases the cell survival. Percent survivals are represented for each indicated genotype treated with talazoparib alone, AZD6738 alone, and the combination of talazoparib and AZD6738. The experiments were performed in triplicate with two independent experiments. Averages and standard deviations are displayed.

**Figure 6:**
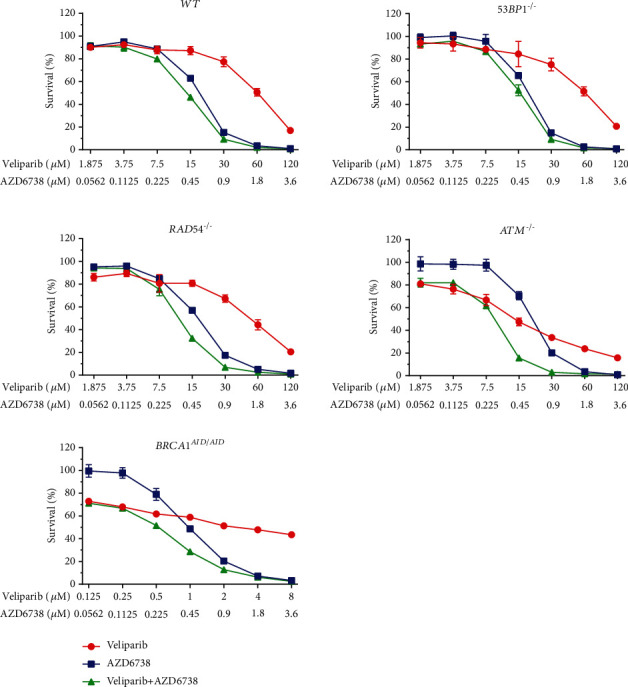
The combination of veliparib and AZD6738 decreases the cell survival. Percent survivals are exhibited for each indicated genotype exposed to veliparib and/or AZD6738. The experiments were performed in triplicate with two independent experiments. Averages and standard deviations are shown.

**Figure 7:**
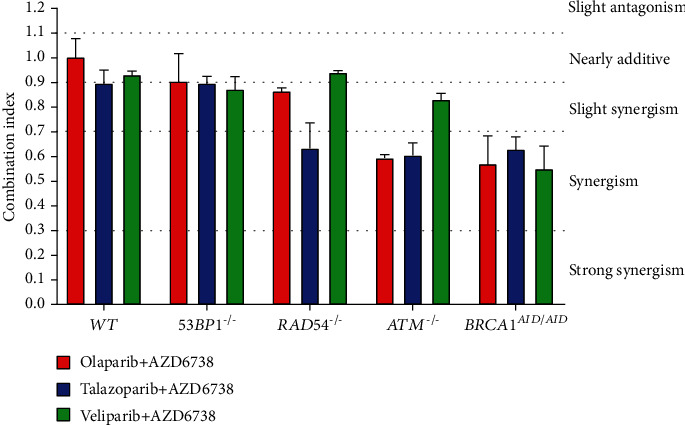
AZD6738 synergizes PARP inhibitor cytotoxicity in specific cell lines. CI values were calculated at IC_50_ of PARP and ATR inhibitors of indicated genotypes using CompuSyn version 1.0. CI < 3 indicates strong synergism; 0.3 < CI < 0.7 indicates synergism; 0.7 < CI < 0.9 indicates slight synergism; 0.9 < CI < 1.1 indicates additive effects; CI > 1.1 indicates antagonism. Averages and standard errors of two independent experiments are displayed.

To investigate the synergistic interaction between PARP and ATR inhibitors, DNA repair-deficient cell lines were treated with serial-diluted concentrations of PARP inhibitor alone, AZD6738 alone, and the combination of PARP inhibitor and AZD6738 (Figures 4–6). CI values were calculated for each combination. According to the results of drug sensitivity assays and IC_50_ estimation, talazoparib is the most potent PARP inhibitor when compared to olaparib and veliparib, while veliparib is the least potent. With respect to deficiencies in specific DNA repair proteins, the *BRCA1^AID/AID^* cell line was the most sensitive cell line in response to PARP inhibitors. The *ATM^−/−^* cell line came second followed by the *RAD54^−/−^* cell line, but the *53BP1^−/−^* cell line was not clearly sensitive to PARP inhibitors (Supplementary Table S1). Additionally, IC_50_ values for AZD6738 of each DNA repair-deficient cell line were not considerably different when compared to *WT* (Supplementary Table S1). Using CI, the synergistic interaction between talazoparib and AZD6738 was observed in *RAD54^−/−^* (CI = 0.63 ± 0.10), *ATM^−/−^* (CI = 0.605 ± 0.05), and *BRCA1^AID/AID^* cell lines (CI = 0.63 ± 0.06). For the combination of olaparib and AZD6738, the synergistic interaction was observed in both *ATM^−/−^* (CI = 0.59 ± 0.01), and *BRCA1^AID/AID^* cell lines (CI = 0.57 ± 0.12), while combining veliparib with AZD6738 showed the synergy in only the *BRCA1^AID/AID^* cell line (CI = 0.55 ± 0.10) (Figure 7). These results suggest that the synergistic interaction of the different combinations of PARP inhibitors and AZD6738 is exclusive for some specific DNA repair-deficient cells.

## 4. Discussion

Nowadays, several PARP inhibitors have been approved for cancer patients. However, most cancer patients who are prescribed the PARP inhibitor have to have germline mutations in *BRCA1/2* genes. In addition to *BRCA1/2*, several somatic mutations in genes associated with the HRR pathway, such as *ATM*, *BAP1*, and *PALB2* genes, have been recognized [39]. Both replication stress and replication-associated DNA damage activate the cell cycle checkpoint through the ATR/CHK1 signaling pathway [40]. In addition to DDR, ATR also promotes the interaction between BRCA1 and PALB2 and the localization of PALB2 to dsDNA breaks that require rapid and efficient repair mechanisms, such as HRR to repair [41]. Previously, the combination of PARP and ATR inhibitors has been investigated in ATM- and BRCA-deficient cells [20, 21]. The inhibition of the other intact DNA repair pathways is a potential strategy to expand the use of PARP inhibitors to treat cancers that are defective in DNA repair genes beyond *BRCA1/2*. However, the impacts of DNA repair dysfunction on response to the combination of PARP and ATR inhibitors are not fully understood.

This study showed that AZD6738 abolishes the G2/M checkpoint activated by PARP inhibitors, leading to the increases in both micronuclei and DNA damage that is consistent with a previous study [42]. Though the PARP inhibitor-induced G2/M arrest which is rescued by AZD6738 is not dependent on status of specific DNA repair genes, the greater increases in micronuclei and DNA lesions are observed in RAD54-, ATM-, and BRCA1-deficient cell lines compared to *WT* cell lines when combining each individual PARP inhibitor with AZD6738. It indicates that AZD6738 tends to potentiate PARP inhibitors in HRR-deficient cells. A significantly increased number of micronuclei at basal levels in RAD54-, ATM-, and BRCA1-deficient cell lines, which show the synergistic interaction between some types of PARP inhibitors and AZD6738, are also observed. However, in a 53BP1-deficient cell line, a number of micronuclei at basal levels are significantly elevated (Supplementary Table S2). It suggests that a number of micronuclei at basal levels would not be a reliable biomarker to predict the response to the combination of PARP and ATR inhibitors. In contrast to RAD51, an accumulation of RAD51 at DNA damage sites directly reflects the activity of the HRR pathway [43]. Thus, RAD51 could be a more precise marker than micronuclei to predict the synergism of PARP and ATR inhibitors.

This study showed novel opportunities for PARP inhibitors by combining with DDR inhibitors in DNA repair-deficient cell lines. Mechanistically, the synergistic interaction of AZD6738 combined with PARP inhibitors is similar to a previous study [44]. Although the increases in micronuclei and dsDNA breaks are observed in HRR-deficient cells when simultaneously treated with PARP inhibitors and AZD6738, the calculated parameter like CI is required to indicate the synergism. Our results are consistent with the previous study that ATR inhibitors enhanced the anticancer activity of PARP inhibitors [44]. Interestingly, the synergistic interaction of different combinations of PARP inhibitor and AZD6738 is specifically observed in some DNA repair-deficient cell lines. Combining olaparib with AZD6738 is synergistic in ATM- and BRCA1-deficient cell lines, but not in *WT*, 53BP1-, and RAD54-deficient cell lines. Moreover, the combination of talazoparib and AZD6738 shows the synergistic interaction in RAD54-, ATM-, and BRCA1-deficient cell lines except *WT* and 53BP1-deficient cell lines. However, veliparib in combination with AZD6738 is exclusively synergistic in a BRCA1-deficient cell line. This finding suggests that cancers with DNA repair deficiency due to the mutations in HRR pathway-associated genes apart from *BRCA1/2* could synergistically respond to the specific combinations of PARP and ATR inhibitors, and the combination strategy of PARP and DDR inhibitors could probably be expanded to treat other DNA repair-deficient cancers besides BRCA1/2-deficient cancers. Nevertheless, the reasons why some combinations of PARP inhibitors and AZD6738 show the synergism in specific HRR-deficient cell lines are unclear. For example, talazoparib, which is 100-fold more potent than olaparib, while veliparib is the weakest one [45], shows the synergistic interaction with AZD6738 in three DNA repair-deficient cell lines. The synergisms of veliparib and olaparib are found in one and two DNA repair-deficient cell lines, respectively, when combining with AZD6738. It might be due to the different abilities of PARP inhibitors to trap PARP onto DNA.

This study also showed that there is no considerable difference in IC_50_ values of AZD6738 among different genotypes, even though an increased number of micronuclei were noticed in DNA repair-deficient cell lines, especially in a BRCA1-deficient cell line. According to the reported IC_50_ values of AZD6738 from this study (Supplementary Table S1), it implicates that AZD6738 does not target HRR-deficient cells; however, a previous study has reported that AZD6738 preferentially targets HRR-deficient tumor cells [46]. It might be due to the shorter time of drug exposure in this study. ATR inhibition does not directly generate dsDNA breaks, but it allows dsDNA breaks to accumulate and induce cell death eventually. To reveal the differential sensitivity of HRR-deficient cells in response to AZD6738, sensitivity assays may require more rounds of cell division to pile up DNA lesions to kill the cells.

For a clinical implication, the synergism demonstrated in this study supports the rationale to further investigate the combination of PARP and ATR inhibitors in HRR-deficient cells beyond BRCA1/2 that may give advantages for cancer patients without germline mutations in *BRCA1/2*. Moreover, PARP inhibitor dosages, especially talazoparib whose side effects are nearly similar to conventional chemotherapeutic drugs [39], could be lowered with the same anticancer activity in combination with AZD6738, resulting in reduced adverse side effects of PARP inhibitors. Nonetheless, combining PARP inhibitors with ATR inhibitors may increase collateral damage to normal cells. Therefore, to minimize the side effects of the combination of PARP and ATR inhibitors, further investigation in a clinical trial to evaluate the safety and efficacy in cancer patients is required.

## 5. Conclusion

In conclusion, this work emphasizes the value of investigating the possible indications of the combination of PARP and ATR inhibitors for cancer treatments to enhance the efficacy of PARP inhibitors and expand their use to other groups of cancer patients. This work demonstrates the evidence that supports the development of cancer therapy through the combination strategy of PARP and ATR inhibitors, and assesses HRR deficiency as predictive biomarkers to identify individuals tending to respond to PARP and ATR inhibitor combination treatments. The use of DDR inhibitors to synergistically potentiate PARP inhibitors could be an alternative option for cancer patients in the future.

## Data Availability

Raw data are available from the corresponding author upon reasonable request.

## Supplementary Materials

Supplementary Materials Supplementary Table S1: IC_50_ values for olaparib, talazoparib, veliparib, and AZD6738 of indicated genotypes were estimated by CompuSyn version 1.0. Supplementary Table S2: a number of micronuclei at the basal levels of indicated genotypes.
