# Supplementary material for: ATR Inhibitor Synergizes PARP Inhibitor Cytotoxicity in Homologous Recombination Repair Deficiency TK6 Cell Lines
Source: Biomed Res Int. 2023 Feb 6;2023:7891753. doi: 10.1155/2023/7891753 (PMC9925244; doi:10.1155/2023/7891753)
Supplement: Supplementary Materials — Supplementary Table S1: IC50 values for olaparib, talazoparib, veliparib, and AZD6738 of indicated genotypes were estimated by CompuSyn version 1.0. Supplementary Table S2: a number of micronuclei at the basal levels of indicated genotypes. [file 7891753.f1.docx]

SUPPLEMENTARY TABLE S1: IC_50_ values for olaparib, talazoparib, veliparib, and AZD6738 of indicated genotypes were estimated by CompuSyn Version 1.0. Averages and standard errors of at least two independent experiments are reported.

| Drug | Genotype | IC_50_ (µM, average±standard error) |
| --- | --- | --- |
| olaparib | *WT* | 13.59075±1.96265 |
|  | *53BP1^-/-^* | 8.72803±0.46658 |
|  | *RAD54^-/-^* | 3.12683±0.20569 |
|  | *ATM^-/-^* | 1.14420±0.14660 |
|  | *BRCA1^AID/AID^* | 0.08888±0.00012 |
| talazoparib | *WT* | 0.10052±0.00663 |
|  | *53BP1^-/-^* | 0.19298±0.01426 |
|  | *RAD54^-/-^* | 0.01897±0.00921 |
|  | *ATM^-/-^* | 0.00498±0.00124 |
|  | *BRCA1^AID/AID^* | 0.00108±0.00006 |
| veliparib | *WT* | 48.94245±8.46285 |
|  | *53BP1^-/-^* | 58.59120±0.90880 |
|  | *RAD54^-/-^* | 45.83755±0.75745 |
|  | *ATM^-/-^* | 16.72290±2.54390 |
|  | *BRCA1^AID/AID^* | 2.34612±0.59620 |
| AZD6738 | *WT* | 0.42157±0.02508 |
|  | *53BP1^-/-^* | 0.39930±0.03101 |
|  | *RAD54^-/-^* | 0.40932±0.02528 |
|  | *ATM^-/-^* | 0.45164±0.04024 |
|  | *BRCA1^AID/AID^* | 0.41537±0.04742 |

SUPPLEMENTARY TABLE S2: A number of micronuclei at the basal levels of indicated genotypes. Final concentration of DMSO in culture media was lower than 0.1%. Averages and standard deviations from at least nine independent experiments are reported. The statistical difference of a number of micronuclei between *WT* and each individual genotype was calculated using Student’s *t*-test. ****p*<0.001; *****p*<0.0001.

| Genotype | A number of micronuclei per cell (average±standard deviation) |
| --- | --- |
| *WT* | 0.0120±0.0051 |
| *53BP1^-/-^* | 0.0366±0.0122**** |
| *RAD54^-/-^* | 0.0214±0.0057*** |
| *ATM^-/-^* | 0.0262±0.0105*** |
| *BRCA1^AID/AID^* | 0.0686±0.0146**** |
